# Parents Know Best: Uncovering a Rare Allergy During Anesthesia Consultation

**DOI:** 10.1155/crpe/4314186

**Published:** 2024-12-26

**Authors:** Charlotte Thirion, Françoise Pirson, Mona Momeni

**Affiliations:** ^1^Department of Anesthesiology, Cliniques Universitaires Saint Luc, Bruxelles, Belgium; ^2^Department of Pneumology, Cliniques Universitaires Saint Luc, Bruxelles, Belgium

**Keywords:** allergy, anesthesiology, atropine, case report, pediatric anesthesia

## Abstract

A 17-month-old child presented for an anesthesia consultation before planned plagiocephaly correction one week later. The medical history by the mother reported an episode of facial redness after administering atropine-based eye drops when the child was 9 months old. Based on this information, the anesthesiologist decided to postpone the surgery and conduct an allergy assessment. Skin tests performed by a pneumo-allergologist in a hospital setting were positive for atropine. Atropine, a frequently utilized drug in anesthesiology, is rarely associated with allergic reactions, particularly in pediatric patients, as evidenced by poor prior descriptions. This case report underscores the pivotal role of preoperative anesthesia consultations and the significance of attentively considering parental perspectives. Allergy testing can be fastidious in infants, and postponing surgery can be challenging, but in our case, it allowed for a safe procedure and no adverse reactions.

## 1. Introduction

Atropine is a widely used anticholinergic medication in anesthesia, especially in children who tends to experience more bradycardia episodes during inhalation anesthesia. Atropine allergy is extremely rare and not well known by anesthesiologists. We describe a case of a child who presented a cutaneous reaction to atropine-containing drops. At preoperative visit, the anesthesiologist in charge of the patient postponed the surgery and requested allergy skin tests. This decision based on the anamnesis with the parents altered the child's preoperative management and allowed the procedure to take place in a more secure situation.

## 2. Case Report

A 17-month-old child presented at a preoperative anesthesia consultation for elective craniosynostosis surgery. The medical history revealed a healthy child, except for an adverse event after a first eye-drop administration a few months earlier. Atropine 0.5% eye drops for strabismus had been administered at home. Shortly after administration, the child developed significant facial redness with mild swelling. There were no signs of systemic allergy. The child had been brought to the emergency department, but by that time the symptoms had spontaneously disappeared, and a possible overdose was considered. The parents were advised not to use the eye drops again. Based on this unusual anamnesis, the anesthesiologist decided to postpone the surgery and requested allergy skin tests.

A skin test was performed by a pneumo-allergologist in a hospital setting under hemodynamic surveillance. The reference values for the skin tests were 0 mm for the negative control prick test, a papule of 5.5 mm in largest diameter for the positive control prick test (histamine) and 4 mm for the negative control intradermal test.

The Atropine Alcon 1% resulted in a strongly positive prick test (6-mm papule) and a positive intradermal reaction at 1/1000 (8.5-mm papule), despite a very low injected volume (< 0.02 mL). Atropine sulfate 0.5 mg/mL was also tested, with a negative prick test, a negative intradermal reaction at 1/1000, but a doubtful reaction at 1/100 and a positive reaction at 1/10, with a 7-mm papule diameter (despite an injected volume of < 0.02 mL). The reaction to atropine sulfate means that the reaction cannot be attributed to an excipient.

A prick test with pure Atropine Alcon 1% eye drops was conducted on two healthy control subjects, and it remained negative in both cases.

Due to the technical difficulties in conducting intradermal reactions in a conscious toddler, only atropine was tested. The patient's parents were given an allergy card. The pneumo-allergologist recommended excluding scopolamine and glycopyrrolate from anesthesia practice due to their similar chemical formula.

The surgery was successfully conducted 2 months later under general anesthesia. Inhalational induction without premedication was performed. Ephedrine, a sympathomimetic agent, was available in case an episode of bradycardia would occur during induction. During the procedure, complete avoidance of atropine and scopolamine was ensured. Isoproterenol was on hand. Other anesthetic drugs most commonly responsible for allergies were administered with caution or not used. A test dose was administered before giving antibiotics. Neuromuscular blocking agents (NBAs) were not used.

## 3. Discussion

Atropine use is extensively described in the treatment of bradycardia with hemodynamic instability, in cases of cholinesterase inhibitor poisoning or intoxication to medications that cause bradycardia. It is also used as a premedication before general anesthesia to reduce vagal reflexes and to decrease hypersalivation and pulmonary secretions especially during pediatric anesthesia induction. However, this practice is becoming less common [1]. Nevertheless, the Pediatric Advanced Life Support Guideline still recommends the use of atropine prior to intubation in specific emergency situations when there is a higher risk of bradycardia.

A literature search reveals that hypersensitivity to atropine is rare, with few reported cases in the literature [2–7] and only one case in a pediatric patient [8]. Immediate hypersensitivity to atropine (iv or ophthalmic administration) is extremely rare, and allergologic investigation is not systematically performed to document an IgE immunologic reaction [9]. The most common causes of intraoperative anaphylaxis are NBA, antibiotics, disinfectants, and latex [10], and in our case, it was used with caution.

The role of the anesthesiologist during pediatric preoperative consultation is challenging because parents often mix up common drug side effects or intolerances as immune-mediated allergies. Nevertheless, they know their child the best and often observe subtle abnormalities that may be overlooked by a physician. Contrary to literature articles advising to reassure parents and demystify allergies, we recommend taking into account the medical history, even if it means postponing surgery for further evaluation [11].

Given the dramatic consequences that an anaphylactic shock can have, it is important to detect an eventual allergy to commonly used intraoperative drugs. The preoperative consultation is an opportunity for referral for allergy testing [11], and it can have a life-changing impact on the anesthetic management of a patient. The agent responsible for the allergy remains of unknown origin in 30% to 50% of cases despite evaluation. The testing of this infant will allow to have a precise diagnosis that will prevent an allergic reaction to atropine for the rest of her life. A close collaboration between surgeons, anesthesiologist, and pneumo-allergologist is mandatory for the safety of the patients with an allergy suspicion [10].

Communication problems are at the roots of medical error. Therefore, it is crucial that the anesthesiologist in charge of the patient informs all the caregivers of the child's allergy throughout the child's perioperative journey. An adapted anesthesia plan must be established and shared with all caregivers in order to prevent accidents [11, 12].

## 4. Conclusion

Hypersensitivity to atropine, although rare, does exist. Preoperative anesthesia consultation is crucial, and gathering a thorough medical history from parents is of utmost importance. When hypersensitivity to commonly used intraoperative drugs is discovered, an appropriate anesthesia plan should be established and shared with the operating room staff. Close collaboration and communication between the surgical and anesthesiology teams is essential for patient safety.

## Data Availability

All data underlying the results are available as part of the article.
